# The Thoroughbred Theory: Influence of Breed on Performance at the CCI5*-L Level of Eventing

**DOI:** 10.3390/ani15121796

**Published:** 2025-06-18

**Authors:** Kianna R. Walz, Meghan E. McCormick, Carleigh E. Fedorka

**Affiliations:** 1 Department of Animal Sciences, Colorado State University, Fort Collins, CO 80523, USA; kianna.walz@colostate.edu; 2 Department of Pharmacy Practice, University of Rhode Island, Kingston, RI 02881, USA; meghanmccormick@uri.edu

**Keywords:** Thoroughbred, eventing, CCI5*-L, horse, warmblood

## Abstract

Breed bias exists within many of the top disciplines of equestrian sport. Therefore, the objective of this study was to evaluate the influence of breed on performance at the CCI5*-L level of eventing. To do so, the results of all CCI5*-L events from 2014 to 2024 were assessed. Initially, Thoroughbreds were compared to all non-Thoroughbreds, and this was followed by assessing the ten primary breeds. Data included penalties accrued in dressage, cross country, show jumping, and overall. Additionally, the likelihood of completing each phase was assessed. It was found that Thoroughbreds accrued more dressage penalties than non-Thoroughbreds (*p* < 0.001). When comparing primary breeds, Thoroughbreds were found to accumulate more dressage penalties than Hannoverian, Irish Sport Horse, Dutch Warmblood, Oldenburg, and Selle Francais (*p* < 0.03). When assessing cross-country, Thoroughbreds were more likely to finish without fault when compared to non-Thoroughbreds (*p* < 0.001). Non-Thoroughbreds were more likely to show jump without fault (*p* < 0.001), while Thoroughbred were found to have higher overall show jumping penalties in comparison to Anglo-European Studbook, Holsteiner, Irish Sport Horse, Dutch Warmblood, Oldenburg, and Selle Francais (*p* = 0.01). When evaluating overall penalties accumulated throughout the event, there was no effect of breed (*p* = 0.42). Overall, there was no advantage in competing a non-Thoroughbred in comparison to a Thoroughbred when considering total penalties accrued, indicating a level playing field within competition. Therefore, breed bias against TBs should be negated when selecting for CCI5*-L eventing prospects.

## Simple Summary

Warmbloods are commonly perceived to outperform Thoroughbreds (TB) in many equestrian disciplines, including eventing. Here, we evaluated the impact of breed on performance at the CCI5*-L level of eventing. The results from all CCI5*-L events between 2014 and 2024 were assessed for performance based on breed (TB vs. non-TB) in addition to within primary breeds (Anglo-Arabian, Anglo-European Studbook, Hannoverian, Holsteiner, Irish Sport Horse, Dutch Warmblood, Oldenberg, Selle Francais, and Sport Horse of Great Britain). The impact of breed on dressage penalties, cross country penalties, show jump penalties, and overall penalties were evaluated. Additionally, the likelihood of completing cross country and completing the overall event was assessed. While Thoroughbreds were found to accrue more dressage and show jumping penalties, they out-performed other breeds in the cross country phase of eventing. No impact of breed was noted in likelihood to complete the event, nor overall penalties accrued, indicating a level playing field. Therefore, breed bias against Thoroughbreds should be negated when selecting for CCI5*-L eventing prospects.

## 1. Introduction

Eventing is an equestrian sport that tests the training, athleticism, and stamina of multiple breeds of horses [1]. Competition within this sport begins at the national level and proceeds to the international level, where the culmination of the sport is seen at the five-star level (CCI5*-L). Few horse-and-rider combinations make it to this preeminent level. In fact, when assessing the years 2018–2024, 56,766 entries were noted at the 2* level, 40,073 entries at the 3* level, 21,887 entries at the 4* level, and only 1947 entries at the 5* level [2]. Each year only seven CCI5*-L events take place throughout the world, and these competitions attract horses and riders from a wide range of countries to take on the ultimate test.

A variety of breeds are noted at the top levels of this equestrian sport, with the majority being of either warmblood or Thoroughbred lineage. The genetics of high performance within this sport have limited data to support breed bias, and yet favoritism exists [3,4]. Due to the stamina required for successful completion of all three phases, an increase in Thoroughbred blood percentage (the amount of Thoroughbred genetics within the offspring) has been pursued at the upper levels of sport [5,6]. Full Thoroughbreds are commonly perceived to perform poorly in comparison to warmbloods at the top level of this sport. Therefore, various warmblood breeds are represented by high entries in the eventing discipline; this includes Irish Sport Horse, Selle Francais, and Trakehner, in addition to various breeds deriving from Germany, including Hanoverian, Oldenburg, and Holsteiner [7]. Warmbloods are believed to outperform the Thoroughbred in the dressage and show jumping phases due to genetic selection for these disciplines [8,9,10,11,12,13,14]. The classic long format of eventing, which included a steeplechase portion, favored the speed and high endurance seen in Thoroughbreds [7,15] and an increased number of Thoroughbred stallions represented in warmblood horses was noted during this era [16]. The long format was eliminated in 2005, and this coincided with an increase in the difficulty of the dressage and show jumping phases, potentially favoring horses selectively bred for these disciplines over the Thoroughbred. Other studies have looked at all traits of horses in eventing to evaluate the best combination of factors for success [7], but none have specifically focused on breed. Additionally, while breed may influence overall outcomes of events, other variables may be of greater impact, including experience of competitor, experience of horse, conditions of the day, and subjective opinion of judging.

Although public perception favors specific breeds and genetics, there is minimal data to support the correlation between breed and performance in the eventing discipline. Therefore, we aimed to evaluate the relationship between breed and performance at the CCI5*-L level of eventing, with a key focus on dressage, cross-country, show jumping, and overall penalties. We hypothesized that breed will not impact the overall performance of the horse, and that the bias against Thoroughbreds will be unfounded.

## 2. Materials and Methods

### 2.1. Overall Data Entry

In this study, the results of all CCI5*-L (previously 4*L) events were collected from 2014 to 2024. This included Adelaide (2014–2019; 2023–2024), Badminton (2014–2019; 2022–2024), Burghley (2014–2019; 2022–2024), Kentucky (2014–2019; 2021–2024), Luhmuhlen (2014–2019; 2021–2024), Maryland (2022–2024), and Pau (2014–2024). The majority of events did not take place in 2020 or 2021 due to the COVID-19 global pandemic. A total of 2696 results were acquired, representing 71 breeds.

In the first analysis, participants were grouped as Thoroughbred (TB; *n* = 330) or non-Thoroughbred (*n* = 2366). Comparisons were made regarding likelihood to complete, likelihood to complete cross country without jump penalties, likelihood to complete cross country without jump or time penalties, likelihood to complete show jumping without jump penalties, and likelihood to complete show jumping without jump or time penalties. Additionally, the total number of penalties noted in dressage, cross country, show jumping, and overall were compared between TB and non-TB. Dressage and overall penalties were also assessed as prior to the removal of the 1.5 dressage coefficient (2014–2017) or after (2018–2024).

Breeds were then grouped as primary or secondary based on a cut-off number of forty entries per breed, leaving 2275 results to be analyzed. Primary breeds were further assessed for the number of dressage penalties, the number of cross country penalties, the number of show jumping penalties, and overall accumulated penalties. This was also assessed both in 2014–2017 in addition to 2018–2024 due to the change in dressage scoring coefficient that was implemented in 2018. Representative data for primary breeds can be seen in Table 1.

### 2.2. Statistics

All statistical analyses were performed in SAS 9.4^®^ (SAS Institute, version 12.1.0; Cary NC, USA). All data was assessed for normality and equal variances using a Shapiro–Wilk test and Bartletts test, respectively. The percentage of Thoroughbreds represented prior to the removal of the dressage coefficient (2014–2017 vs. 2018–2024) was assessed using a chi-squared before continuing on with further analysis. Due to not meeting requirements of equal variances, Thoroughbreds were compared to non-Thoroughbreds utilizing a Mann–Whitney *t*-test to assess dressage, cross country, show jumping, and overall penalties. For dressage and overall penalties, this analysis was performed as 2014–2017 in addition to 2018–2024. Additionally, a chi-squared test was used to compare the percentage of Thoroughbreds to non-Thoroughbreds likelihood to finish, likelihood to jump clean in both phases, and likelihood to be faultless in both jumping disciplines. When assessing primary breeds, a Kruskal–Wallis nonparametric one-way ANOVA was utilized to compare medians. Post hoc analysis was performed using a Tukey test for multiple comparisons. Due to data not meeting the requirements of parametric analysis, it is expressed as median ± interquartile range. Significance was set to *p* ≤ 0.05.

## 3. Results

### 3.1. Dressage

**Figure 1:**
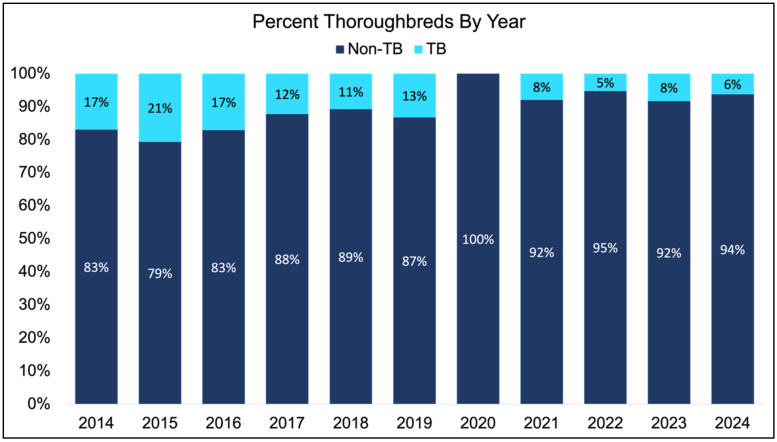
Percentage of Thoroughbreds competing at the FEI CCI5*-L level from 2014 to 2024. A decrease in the number of Thoroughbreds entered at the CCI5*-L level was noted between the years of 2018 and 2024 in comparison to 2014 to 2017.

**Figure 2:**
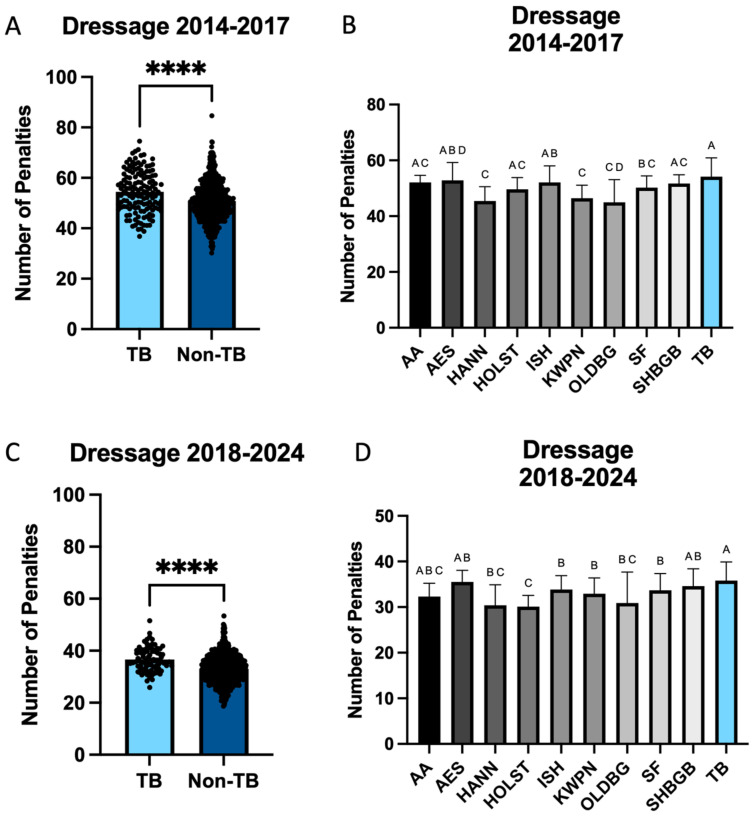
Dressage penalties accumulated in Thoroughbred and non-Thoroughbred breeds at the FEI 5* level. (**A**) Thoroughbreds acquired more penalties than non-Thoroughbreds breeds between the years 2014 and 2017. (**B**) Between 2014 and 2017, this was primarily noted as HANNs and KWPNs accruing the least dressage penalties, while TBs accrued the most. (**C**) Thoroughbreds acquired more penalties than non-Thoroughbreds breeds between the years 2018 and 2024. (**D**) From 2018 to 2024, HOLST accrued the fewer dressage penalties, while TBs continued to accrue the most penalties within this phase. **** *p* < 0.0001. ^A,B,C,D^ indicates significance.

The percentage of Thoroughbreds competing within year was assessed prior to analyzing dressage scores due to the change in penalty coefficient that was implemented in 2018. A significant effect of year was noted (*p* < 0.0001; Figure 1), analysis was performed within 2014–2017 and then again on the years between 2018 and 2024 following the redaction of the coefficient. Non-Thoroughbreds accrued significantly fewer dressage penalties when compared to Thoroughbreds between the years 2014–2017 (50.8 vs. 54.4; *p* < 0.001; Figure 2A). When evaluating the primary breeds represented during this time, significant differences were noted within breed (*p* < 0.001; Figure 2B). The lowest number of dressage penalties was acquired by HANNs (46.45 ± 1.45), and this was found to be significantly lower than penalties acquired by AESs (53.56 ± 1.99; *p* = 0.01), ISH (52.83 ± 1.38; *p* < 0.001), and TB (54.38 ± 1.45; *p* < 0.001). In contrast, TBs were found to have significantly higher dressage penalties in comparison to those accrued by KWPNs (47.18 ± 1.25; *p* < 0.001), OLDBGs (47.18 ± 1.89; *p* < 0.001) or SF (49.89 ± 1.30; *p* = 0.01). Additionally, ISH were found to have significantly more dressage penalties (52.83 ± 1.23) in comparison to KWPNs (47.18 ± 1.18; *p* < 0.001) and OLDBGs (46.45 ± 1.85; *p* = 0.01).

When assessing dressage penalties accrued between the years 2018 and 2024, significant differences were still noted between non-Thoroughbreds and Thoroughbreds (33.3 vs. 36.1; *p* < 0.001; Figure 2C), with non-Thoroughbreds accruing significantly fewer penalties. When comparing primary breeds, a different picture than what was noted during 2014–2017 was observed. HOLSTs acquired the least number of dressage penalties on average (29.94 ± 0.96), and this was significantly lower than AESs (34.88 ± 0.99; *p* < 0.001), ISHs (33.57 ± 0.72; *p* < 0.001), KWPNs (32.83 ± 0.83; *p* = 0.02), SF (33.26 ± 0.88; *p* < 0.01), SHGBGs (34.29 ± 0.98; *p* < 0.001), and TBs (36.53 ± 0.89; *p* < 0.001; Figure 2D). Additionally, TBs acquired significantly more dressage penalties (36.53 ± 0.89) than HANN (31.35 ± 1.10; *p* < 0.001), ISH (33.57 ± 0.65; *p* < 0.001), KWPNs (32.83 ± 0.77; *p* < 0.001), OLDBGs (32.72 ± 1.13; *p* = 0.03), and SF (33.26 ± 0.82; *p* < 0.01).

### 3.2. Cross Country

**Figure 3:**
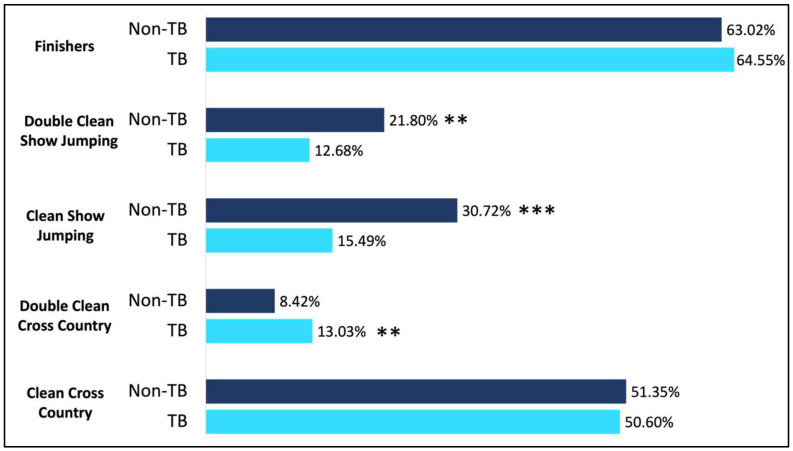
Likelihood to complete at the FEI 5* level. When comparing Thoroughbreds to non-Thoroughbreds, no difference was noted in the likelihood to complete at the CCI5*-L level. With regard to finishing, 64.5% of the Thoroughbreds finished, while 63.02% of the non-Thoroughbred entries finished. Roughly 50% of starters completed cross country without jump penalties. Of these, Thoroughbreds were significantly more likely to be without both jump and time penalty in cross country (13.0%) compared to non-Thoroughbreds (8.4%). With regard to show jumping, 30.7% of non-Thoroughbreds completed this phase without jump penalty, and this was significantly higher than Thoroughbreds. When assessing both jump and time penalties in show jumping, this was reduced to 21.8% of non-Thoroughbreds, which was significantly higher than only 12.7% of Thoroughbreds. ** *p* < 0.01, *** *p* < 0.001.

**Figure 4:**
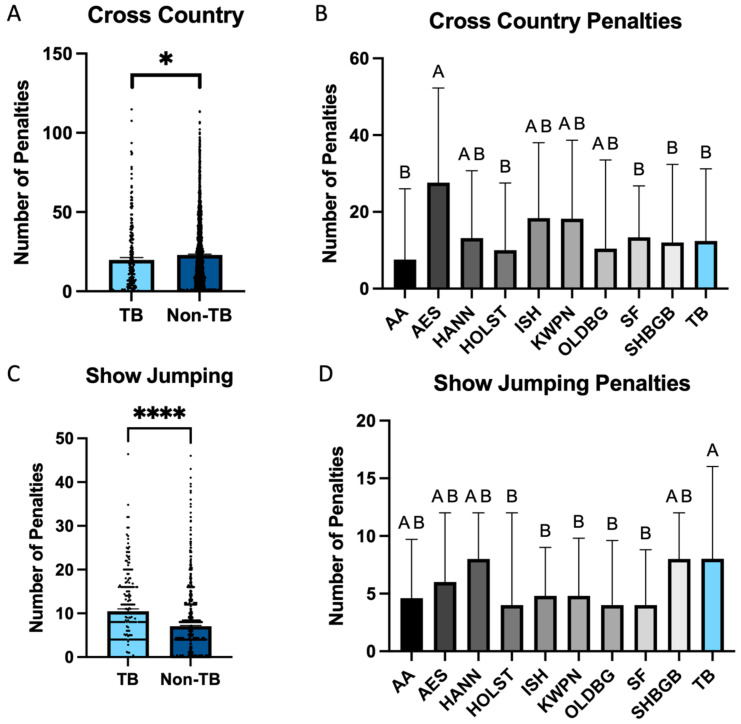
Overall jump penalties accumulated at the FEI 5* level from 2014 to 2024. (**A**) An overall influence of breed was noted when assessing overall cross country penalties, with Thoroughbreds accumulating fewer penalties than non-Thoroughbreds. (**B**) When assessing primary breeds, a significant reduction in overall cross country penalties was noted for AAs, HOLSTs, SFs, SHBGBs, and TBs in comparison to AES. (**C**) When assessing overall show jumping penalties, Thoroughbreds were significantly more likely to acquire penalties than non-Thoroughbreds. (**D**) When assessing primary breeds, this was noted as HOLSTs, ISHs, KWPNs, OLDBGs, and SFs accruing significantly fewer show jump penalties than TBs. * *p* < 0.05, **** *p* < 0.0001. ^A,B^ indicates significance.

When assessing cross country jump faults, there was no effect of breed on the likelihood to have no jump faults on cross country (*p* = 0.78; Figure 3). Zero jump penalties were observed in 1220/2348 of the non-Thoroughbred horses (51.4%), and this was not different than the 166/330 of Thoroughbreds (50.6%) that completed cross country without jump penalty. When assessing both jump and time faults, Thoroughbreds were significantly more likely to be faultless during cross country (*p* < 0.001; Figure 4). Faultless rounds were observed in 46/330 Thoroughbreds (13%), which was significantly higher than in non-Thoroughbreds, where 199/2351 entries were without fault on cross country (8.3%).

There was an effect of breed on the number of total cross country penalties accrued (*p* = 0.05), with Thoroughbreds accumulating fewer cross country penalties than non-Thoroughbreds (19.7 vs. 22.8; Figure 4A). There was also a significant effect of breed on total cross country faults when evaluating primary breeds (Figure 3). This was noted as AAs (13.73 ± 4.9; *p* = 0.02), HOLSTs (18.59 ± 3.52; *p* = 0.02), SFs (17.86 ± 3.24; *p* < 0.01), SHBGBs (20.06 ± 3.32; *p* = 0.04), and TBs (19.86 ± 2.97; *p* < 0.01) having significantly fewer cross country faults when compared to AESs (31.82 ± 2.97). No significant differences were noted when evaluating HANNs, ISHs, KWPNs, or OLDBGs in comparison to any other breed (*p* > 0.05).

### 3.3. Show Jumping

When assessing faults acquired during show jumping, only horses which were found fit to continue during the final veterinary inspection were assessed, leaving 1477 entries. There was no significant effect of breed on the percentage of horses that withdrew following the cross country phase (*p* = 0.91), with 12.2% of Thoroughbreds and 12.4% of non-Thoroughbreds withdrawing following cross country. Of these found fit to compete, 212 were Thoroughbreds, and 1265 were non-Thoroughbreds. Overall, non-Thoroughbreds had significantly less total jump penalties in show jumping compared to Thoroughbreds (7.0 vs. 10.4; *p* < 0.001; Figure 3). Additionally, non-Thoroughbreds were significantly more likely to jump a faultless show jumping round, with 316/1471 (21.48%) non-Thoroughbreds jumping a fault-free round, while only 27/211 Thoroughbreds jumped a fault free round (*p* = 0.003; Figure 3).

When assessing the primary breeds, TBs were found to have significantly higher overall show jumping penalties (10.47 ± 1.46) in comparison to AES (7.67 ± 0.96; *p* = 0.05), HOLSTs (5.85 ± 0.91; *p* < 0.001), ISH (6.70 ± 0.57; *p* < 0.001), KWPNs (6.36 ± 0.75; *p* < 0.001), OLDBGs (6.15 ± 1.14; *p* = 0.01), and SFs (6.00 ± 0.80; *p* < 0.001; Figure 3). No significant differences were noted regarding overall show jumping penalties when comparing TBs (10.47 ± 1.46) to AAs (5.9 ± 1.7; *p* = 0.07), HANNs (8.85 ± 0.98; *p* = 0.91), or SHBGBs (8.11 ± 0.84; *p* = 0.16; Figure 4).

### 3.4. Overall

When assessing the likelihood to complete all three phases, there was no effect of breed (*p* = 0.63), with Thoroughbreds being as likely to complete the event (211/330; 63.94%) as non-Thoroughbreds (1472/2352; 62.59%; Figure 4). The results were again analyzed in 2014–2017 and 2018–2024 due to the influence of the dressage coefficient removal. No significant differences were noted between non-Thoroughbreds and Thoroughbreds in overall penalties within either time frame assessed, and this included 2014–2017 (83.1 vs. 85.4; *p* = 0.42) and 2018–2024 (61.0 vs. 65.7; *p* = 0.12).

**Figure 5:**
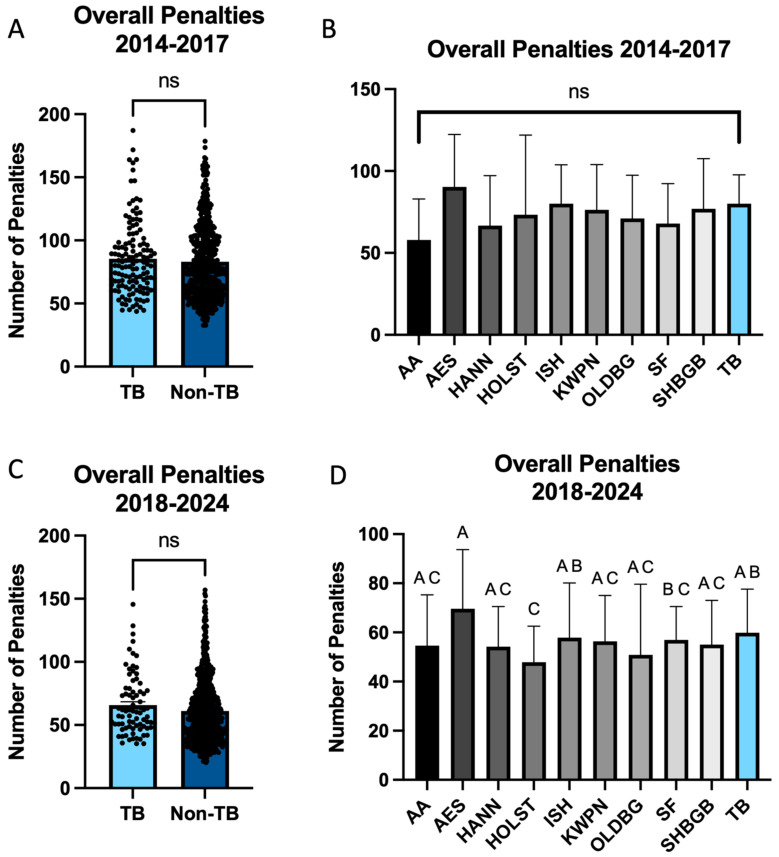
Overall penalties accumulated at the FEI 5* level from 2014 to 2024. (**A**) When assessing 2014–2017, no significant effect of breed was noted when comparing overall penalties accrued by either Thoroughbreds or non-Thoroughbreds. (**B**) This was also noted when assessing primary breeds. (**C**) When assessing 2018–2024, no significant effect of breed was noted when comparing overall penalties accrued by Thoroughbreds or non-Thoroughbreds. (**D**) A significant effect of breed was noted when comparing primary breeds, where HOLST accrued significantly fewer overall penalties in comparison to AES, ISH, and TB. Additionally, AES accrued more penalties than SF. ^A,B,C^ indicates significance, ns = not significant.

When evaluating overall faults with regard to primary breeds, analysis was again performed within 2014–2017 and 2018–2024. Prior to 2018, no significant effect of primary breed was noted on overall penalties accrued (*p* > 0.05; Figure 5A,B). When assessing 2018–2024, a significant effect of primary breed was noted (*p* < 0.001; Figure 5C). This was specifically noted in the AESs, which accumulated significantly more total penalties (72.70) in all three phases than HOLSTs (50.24 ± 9.53; *p* < 0.001) and SF (57.46 ± 7.32; *p* = 0.03; Figure 5D). There were no significant differences noted when comparing HANNs, ISHs, KWPNs, OLDBGs, SHBGBs, or TBs (*p* > 0.05), indicating that none of these primary breeds was more likely to complete an event at the CCI5*-L level with fewer faults than the others. In contrast, HOLSTs had the fewest accrued penalties overall (50.24), and this was significantly lower than ISHs (64.01 ± 6.40; *p* < 0.01) and TB (65.70 ± 6.62; *p* = 0.01).

## 4. Discussion

Selection of genetics for performance purposes has been occurring for centuries. In the horse, performance is heavily biased and can be influenced by objective data (speed, height) or subjective opinion (trainability, jumping ability, and movement). While the priority of genetic selection for Thoroughbreds lies in speed and stamina [17], the majority of warmblood studbooks prioritize jumping ability necessary for high level show jumping and/or movement for advanced dressage [18]. The sport of eventing requires a combination of these genetic aptitudes, and includes the movement for dressage, speed and stamina of cross country, and jumping ability for show jumping. To our knowledge, this is the first study to investigate the influence of breed on performance within the highest level of eventing. 

Dressage is the first phase of a three-day event and requires the execution of specific movements that are judged for precision, cadence, and rhythm. When assessing the results of CCI5*-L within the past decade, Thoroughbreds were found to accrue significantly more dressage penalties when compared to non-Thoroughbreds. The decrease in dressage penalties within the warmblood breeds is not surprising, as many of the breeds have been genetically selected for this phase, such as Holsteiners [19], Dutch Warmblood [13], Hanoverians [20], Selle Francais [21], and Oldenburgs [20]. When assessing Thoroughbreds, the dressage coefficient reduction in 2018 did not appear to impact overall performance, but an impact of this coefficient could be seen when evaluating other primary breeds. Between 2014 and 2017, Hannoverians and KWPNs were found to have the lowest accrued dressage penalties, but this shifted from 2018 to 2024, where Holsteiners were found to have the least dressage penalties during this time. We are unsure as to the cause of this shift, but additional research into blood percentage, sire influence, judging bias [22], and/or rider experience within this comparison is warranted.

In contrast to dressage, the prowess of the Thoroughbred was noted during the second phase of three-day eventing, as Thoroughbreds were more likely to complete cross country without jump or time penalty in comparison to their non-Thoroughbred counterparts. Almost 14% of all Thoroughbred entries at the CCI5*-L level completed cross country penalty-free, and this was in contrast to only 8.3% of non-Thoroughbreds. Although no differences were noted in the percentage of entries that acquired jump penalties within the cross-country phase when comparing Thoroughbreds to non-Thoroughbreds, time penalties appeared to play a pivotal role in cumulative penalties, as Thoroughbreds accrued significantly fewer. Thoroughbreds have been genetically selected for speed and stamina for over 300 years [23,24,25,26,27]. At the CCI5*-L level of eventing, horses are expected to gallop at 570 m per minute for upwards of 6000 m, thereby galloping at speed for 11–12 min; a test for which the genetics of Thoroughbreds align with [23,24,25,28]. For this reason, Thoroughbreds have historically been infused into various warmblood studbooks for the purpose of increasing their speed and stamina, and most European warmblood breeds consist of at least 35% Thoroughbred [28]. Specific breed and breed locale appear to impact blood percentage, as breeds deriving from France have considerably more Thoroughbred blood (47.9%) than those deriving from Germany (31.6%) [28]. The Thoroughbred blood percentage of the non-Thoroughbreds within this study was not evaluated, but this topic deserves future research and is needed to evaluate the relationship between Thoroughbred blood and cross country performance.

When assessing the number of penalties accrued during the show jumping phase of three-day eventing, a strong deviation was noted when comparing Thoroughbreds to non-Thoroughbreds. Non-Thoroughbreds were twice as likely to complete show jumping without jump penalty (30.4% vs. 15.5%, respectively), but when time was considered, this was reduced to a smaller margin (21.8% vs. 12.6%), again indicating the influence of Thoroughbred speed on results. When comparing primary breeds, Holsteiners accrued the least number of penalties in the show jumping phase, with Irish Sport Horse, KPWN, Oldenburg, and Selle Francais being comparable. The Thoroughbred acquired the most show jumping penalties of all primary breeds assessed, but this was not significantly greater than Anglo-Arabians, Anglo-European Studbook, Hannoverians, or Sport Horses of Great Britain. These results are not surprising, as a large portion of the non-Thoroughbreds represented were presumably purpose-bred for show jumping [4,9,10,11,12,13,14,19,21]. Jumping ability is assessed within the inspection process of the vast majority of warmblood studbooks, with the objective being to evaluate a prospects ability to compete at the 1.60 m+ of international show jumping [29]. Due to the strenuous demands of three-day eventing, the maximum height for CCI5*-L show jumping is appreciably lower than this height, with the fences set to 1.30 m. Therefore, it is not surprising that horses purpose bred for 1.60 m+ will outperform a breed that is produced with genetic selection for galloping over flat surfaces.

While a breed difference is noted within the three phases of competition, the cumulative score is of greatest importance. Here, there was no impact of breed on likelihood to complete all three phases, as noted by 64% of Thoroughbreds completing which was not significantly different than the 62.6% of non-Thoroughbreds completing. Additionally, no overall impact of breed was noted within either 2014–2017 or 2018–2024, with Thoroughbreds accumulating similar overall penalties to non-Thoroughbreds. This indicates the heavy influence that cross country has on the overall competition, as the Thoroughbreds competence within the cross-country phase made up for deficits noted following dressage and show jumping. Primary breed did not influence overall penalties in 2014–2017 when the dressage coefficient impacted the influence of dressage, but by removing this coefficient, a significant effect of primary breed was noted. This was observed by Holsteiners accruing fewer penalties than most primary breeds, and this reduction in overall penalties was found to be significant when compared to Anglo-European Studbook, Irish Sport Horses, and Thoroughbreds. Additionally, horses within the Anglo-European Studbook acquired significantly more overall penalties in comparison to Selle Francais. Interestingly, these two specific breeds (Holsteiner and Selle Francais) represent two of the top three warmblood studbooks with regard to Thoroughbred blood percentage, and come in at 45.8% and 52.7%, respectively [30]. Therefore, it may be argued that Thoroughbred blood percentage plays an impactful role on the ability for warmbloods to succeed at the upper levels of eventing, a belief that has always been assumed but never proven. Future research is needed to disseminate the percentage of Thoroughbred within individual studbook for optimal performance.

It is unknown if the specific horses within the present study were purpose-bred for dressage, show jumping, eventing, or even racing, and this presents a limitation of this study. A clear breeding objective is important when selecting intended progeny of any breed. Therefore, it is ideal for breed registries to have specific goals that include all biological traits perceived important for production, both competitively and economically [18]. Breeding for performance is difficult to monitor within a breed registry, as there are diverse desires for performance within studbook, in addition to the level for which the horse is intended to compete (amateur, national, and international). This is particularly difficult for warmblood breeds due to the immense crossbreeding and diverse purposes [29]. A study from 2004 found only eleven of nineteen warmblood studbooks to breed specifically for eventing traits [18]. This was in contrast to sixteen organizations breeding specifically for show jumping, and fourteen breeding specifically for dressage. More recently, a survey was sent to all studbooks within the World Breeding Federation for Sporthorses (WBFSH), and only two of the responding studbooks identified eventing as a breeding objective [29]. In contrast, ten studbooks identified show-jumping and seven studbooks identified dressage as a priority. Overall, eventing is considered a low priority or objective for the majority of warmblood studbooks, and therefore utilizing studbook as an indicator for eventing performance is confounded. The majority of primary breeds represented in the present study are bred with intent to either show jump or do dressage, with specific phenotypes desired for each. The genotypes involved in this selection has been elucidated in Holsteiners [19], KWPNs [13], Hanoverians [20], Selle Francais [21], and Oldenburgs [20], with a marked delineation in the genotype of a successful dressage horse in comparison to show jumper. Additionally, the genetics of these animals differs when attempting to produce either subtype [31]. An additional limitation of this study is that each entry is represented as an individual, when many horses have multiple results within the ten years assessed. Only 1112 horses were represented within the 2696 entries, with an average of 2.4 starts for each horse. This ranged from only a single start at the level to a subset of animals with long careers, including Reve du Rouet (OLDBG; 18 entries), Ringwood Sky Boy (ISH; 15 entries), and LCC Barnaby (ISH; 14 entries), which may lead to a horse influence on overall results. Additionally, the effect of rider and the experience of each rider was not assessed. As entries were assessed over 10 years, the experience of specific riders may have increased over time, influencing the success of individual horses. Additionally, with experience comes increased backing, funding, and promotion, which may lead to purchasing more expensive horses with increased genetic selection. This may lead to rider/breed bias, and future research is needed to investigate the actual Thoroughbred blood percentage, in addition to the influence of experience at the level on final scores.

## 5. Conclusions

In conclusion, bias and negative perception towards the Thoroughbreds’ ability to compete at the CCI5*-L level of eventing should be disregarded. While Thoroughbreds did accrue significantly more penalties in both the dressage and show jumping phase of eventing, their speed, ability, and efficiency within the cross country phase made up for these shortcomings. There was no overall advantage in competing a non-Thoroughbred in comparison to a Thoroughbred when assessing total penalties accrued, indicating a level playing field within competition. Additionally, not all warmblood studbooks were found to be created equal, as only the Holsteiners were significantly more likely to finish a three-day event with fewer penalties than the Thoroughbred. In summary, the decrease in Thoroughbred entries at the 5* level is alarming. We hope that the present study assists in reducing bias when horses are selected as competitive prospects to reach this pinnacle level.

## Figures and Tables

**Table 1:** Summary of data assessed.

Breed	Breed Abbreviation	Number of Entries	Age at Competition (Mean ± SEM)
Anglo-Arabian	AA	43	13.98 ± 0.32
Anglo-European	AES	115	13.08 ± 0.21
Hannoverian	HANN	111	12.57 ± 0.17
Holsteiner	HOLST	126	12.82 ± 0.16
Irish Sport Horse	ISH	864	12.91 ± 0.07
Dutch Warmblood	KWPN	250	12.67 ± 0.11
Oldenburg	OLDBG	71	12.46 ± 0.24
Selle Francais	SF	197	13.08 ± 0.16
Sport Horse of Great Britain	SHBGB	170	13.00 ± 0.16
Thoroughbred	TB	330	13.79 ± 0.13

## Data Availability

Data is available and Forthcoming on Dryad. https://doi.org/10.5061/dryad.bcc2fqzr4.

## Abbreviations

The following abbreviations are used in this manuscript:

AA	Anglo-Arabian
AES	Anglo-European Studbook
HANN	Hannoverian
HOLST	Holsteiner
ISH	Irish Sport Horse
KWPN	Dutch Warmblood
Non-TB	Non-Thoroughbred
OLDBG	Oldenburg
SF	Selle Francais
SHBGB	Sport Horse of Great Britain
TB	Thoroughbred

## References

[1] Bennet E.D., Cameron-Whytock H., Parkin T.D.H. (2023). Federation Equestre Internationale eventing: Fence-level risk factors for falls during the cross-country phase (2008–2018). Equine Vet. J..

[2] O’Connor D., Sinclair G. Risk management data: 2024 Highlights of international statistics. Proceedings of the FEI Eventing Forum.

[3] Ricard A., Chanu I. (2001). Genetic parameters of eventing horse competition in France. Genet. Sel. Evol..

[4] Nazari-Ghadikolaei A., Fikse F., Gelinder Viklund A., Eriksson S. (2023). Factor analysis of evaluated and linearly scored traits in Swedish Warmblood horses. J. Anim. Breed. Genet..

[5] Chance A. (2020). It’s in the Blood: LRK3DE Edition. https://useventing.com/news-media/news/its-in-the-blood-lrk3de-edition.

[6] Chance A. (2023). Blood Percentage: We’ve Been Doing It Wrong. https://eventingnation.com/blood-percentage-weve-been-doing-it-wrong/.

[7] Pietrzak S., Prochniak T., Osinska K. (2013). The influence of certain factors on the results obtained by horses classified in eventing ranks of the International Federation for Equestrian Sports. Ann. Univ. Mariae Curie-Sklodowska Lub.-Pol..

[8] Chapard L., Meyermans R., Gorssen W., Hooyberghs K., Meurrens I., De Smet S., Buys N., Janssens S. (2024). Early life jumping traits: Are they good proxies for success in show jumping competitions in Belgian warmblood horses?. J. Anim. Breed. Genet..

[9] Chapard L., Van Thillo A., Meyermans R., Gorssen W., Buys N., Janssens S. (2023). Adjusted fence height: An improved phenotype for the genetic evaluation of show jumping performance in Warmblood horses. Genet. Sel. Evol..

[10] Prochniak T., Kasperek K., Knaga S., Rozempolska-Rucinska I., Batkowska J., Drabik K., Zieba G. (2021). Pedigree Analysis of Warmblood Horses Participating in Competitions for Young Horses. Front. Genet..

[11] Ablondi M., Eriksson S., Tetu S., Sabbioni A., Viklund A., Mikko S. (2019). Genomic Divergence in Swedish Warmblood Horses Selected for Equestrian Disciplines. Genes.

[12] Rovere G., Madsen P., Norberg E., van Arendonk J.A., Ducro B.J. (2015). Effect of specialization on genetic parameters of studbook-entry inspection in Dutch Warmblood horses. J. Anim. Breed. Genet..

[13] Rovere G., Ducro B.J., van Arendonk J.A., Norberg E., Madsen P. (2017). Genetic correlations between dressage, show jumping and studbook-entry inspection traits in a process of specialization in Dutch Warmblood horses. J. Anim. Breed. Genet..

[14] Rovere G., Ducro B.J., van Arendonk J.A., Norberg E., Madsen P. (2016). Analysis of competition performance in dressage and show jumping of Dutch Warmblood horses. J. Anim. Breed. Genet..

[15] Craig L., Hintz H.F., Soderholm L.V., Shaw K.L., Schryver H.F. (1985). Changes in blood constituents accompanying exercise in polo horses. Cornell Vet..

[16] Bonow S., Eriksson S., Thoren Hellsten E., Gelinder Viklund A. (2023). Consequences of specialized breeding in the Swedish Warmblood horse population. J. Anim. Breed. Genet..

[17] Bailey E., Petersen J.L., Kalbfleisch T.S. (2022). Genetics of Thoroughbred Racehorse Performance. Annu. Rev. Anim. Biosci..

[18] Koenen E.P.C., Aldridge L.I., Philipsson J. (2004). An overview of breeding objectives for warmblood sport horses. Livest. Prod. Sci..

[19] Engel L., Becker D., Nissen T., Russ I., Thaller G., Krattenmacher N. (2022). Mitochondrial DNA Variation Contributes to the Aptitude for Dressage and Show Jumping Ability in the Holstein Horse Breed. Animals.

[20] Nolte W., Thaller G., Kuehn C. (2019). Selection signatures in four German warmblood horse breeds: Tracing breeding history in the modern sport horse. PLoS ONE.

[21] Brard S., Ricard A. (2015). Genome-wide association study for jumping performances in French sport horses. Anim. Genet..

[22] Rovere G., Madsen P., Norberg E., Van Arendonk J.A., Ducro B.J. (2014). Genetic connections between dressage and show jumping in Dutch Warmbloods. Acta Agric. Scand..

[23] Wolframm I. (2023). Let Them Be the Judge of That: Bias Cascade in Elite Dressage Judging. Animals.

[24] Kis J., Rozsa L., Husveth F., Zsolnai A., Anton I. (2021). Role of genes related to performance and reproduction of Thoroughbreds in training and breeding—A review. Acta Vet. Hung..

[25] Sharman P., Wilson A.J. (2023). Genetic improvement of speed across distance categories in thoroughbred racehorses in Great Britain. Heredity.

[26] Han H., McGivney B.A., Farries G., Katz L.M., MacHugh D.E., Randhawa I.A.S., Hill E.W. (2020). Selection in Australian Thoroughbred horses acts on a locus associated with early two-year old speed. PLoS ONE.

[27] Hill E.W., Gu J., Eivers S.S., Fonseca R.G., McGivney B.A., Govindarajan P., Orr N., Katz L.M., MacHugh D.E. (2010). A sequence polymorphism in MSTN predicts sprinting ability and racing stamina in thoroughbred horses. PLoS ONE.

[28] de Oliveira Padilha D.A., Padilha S.F., Martins R., Scheffer B.E.M., Miliorini M.R., Dias L.T., Teixeira R.A. (2025). Estimation of genetic parameters for racing time and ranking in Thoroughbred horses. J. Equine Vet. Sci..

[29] Roman-Popovici A., Constantin S.D., GIlca I. (2014). Study regarding the percentage of English Thoroughbred blood in the last five generations for the best jumping horses in the world. Czech J. Food Sci..

[30] Doyle J.L., Carroll C.J., Corbally A.F., Fahey A.G. (2022). An overview of international genetic evaluations of show jumping in sport horses. Transl. Anim. Sci..

[31] Sobotkova E., Mikule V., Kuritkova D., Jisrova I., Sladek L. (2022). Analysis of the current situation in international show jumping and assessment of the influence of the proportion of Thoroughbred in the pedigree, horse demographics and sport season on the performance of horses. J. Vet. Behav..

